# Obesity Severity Differentially Shapes Diabetes-Related Impairment in Cardiorespiratory Fitness: A Cross-Sectional Propensity Score–Weighted Analysis of Middle-Aged Adults

**DOI:** 10.14740/jocmr6519

**Published:** 2026-05-31

**Authors:** Yan Jin, Ke Liu, Yang Yang, Lu Lu Wang, Zhi Yuan Zhang, Xin Yan Li, Ning Zhang

**Affiliations:** a Department of Rehabilitation Medicine, Nanjing Drum Tower Hospital, Clinical College of Nanjing University of Chinese Medicine, Nanjing 210008, China; b Department of Rehabilitation, College of Acupuncture and Moxibustion and Massage Health, Preservation and Rehabilitation, Nanjing University of Chinese Medicine, Nanjing 210023, China; c Department of Rehabilitation, Nanjing Drum Tower Hospital, Affiliated Hospital of Medical School, Nanjing University, Nanjing, China; d Department of Cardiology, Taikang Xianlin Drum Tower Hospital, Affiliated Hospital of Medical School, Nanjing University, Nanjing, China; e NHC Contraceptives Adverse Reaction Surveillance Center, Jiangsu Health Development Research Center, Nanjing 210008, China; f Department of Ultrasound, Nanjing Drum Tower Hospital, the Affiliated Hospital of Nanjing University Medical School, Nanjing 210008, China; g They authors contributed equally to this study.

**Keywords:** Cardiorespiratory fitness, Cardiopulmonary exercise testing, Obesity, Type 2 diabetes mellitus, Propensity score weighting

## Abstract

**Background:**

Obesity and type 2 diabetes mellitus (T2DM) are each linked to reduced cardiorespiratory fitness (CRF), but it remains unclear whether diabetes-related impairment in exercise capacity depends on obesity severity. This question has been rarely addressed in adults aged 20–50 years, a group in whom obesity and T2DM frequently coexist while advanced cardiovascular complications may still be subclinical.

**Methods:**

In this cross-sectional study, we performed a propensity score–weighted analysis of 823 adults aged 20–50 years who underwent cardiopulmonary exercise testing (CPX). Participants were classified by obesity severity (body mass index (BMI) 24 to < 35 vs. ≥ 35 kg/m^2^) and T2DM status to define four obesity–diabetes phenotypes. We compared peak oxygen uptake (peak VO_2_) and related CPX indices across phenotypes and explicitly tested for a BMI × T2DM interaction. Associations with body composition and glycemic measures were further evaluated using weighted correlation and regression analyses.

**Results:**

After weighting, obesity severity accounted for the largest differences across most CPX indices. Peak VO_2_ showed a significant BMI × T2DM interaction (P for interaction < 0.001): diabetes-associated reductions were evident in moderate obesity (24 to < 35 kg/m^2^) but were markedly attenuated in severe obesity (≥ 35 kg/m^2^). Lean body mass was the strongest independent determinant of peak VO_2_, while 2-h postprandial glucose (but not fasting glucose) was independently and inversely associated with aerobic capacity among participants with T2DM.

**Conclusions:**

In middle-aged adults, obesity severity modifies the association between T2DM and CRF. Body composition and postprandial glycemia, rather than diabetes status alone, appear to be central determinants of aerobic capacity. Together, these results support phenotype-informed interpretation of CPX and motivate stratified approaches to cardiopulmonary assessment and intervention in obesity and T2DM.

## Introduction

Overweight and obesity have risen sharply worldwide over recent decades and now represent a major public health challenge. Obesity is now recognized as a chronic, relapsing disease with complex pathophysiology and substantial cardiometabolic consequences that extend beyond excess caloric intake alone [1]. The coexistence of obesity and type 2 diabetes mellitus (T2DM) is common in clinical practice and carries important consequences for cardiovascular health and functional capacity.

Cardiorespiratory fitness (CRF) reflects integrated cardiovascular, pulmonary, and skeletal muscle function. Low CRF is among the strongest predictors of cardiovascular morbidity and all-cause mortality, and recent reviews continue to support its role as a clinically important integrative marker of cardiometabolic health [2, 3]. Beyond traditional risk factors, low CRF is closely associated with metabolic dysfunction, insulin resistance, and adverse cardiovascular outcomes [4]. Cardiopulmonary exercise testing (CPX) provides a comprehensive and reproducible assessment of CRF by jointly capturing oxygen uptake, ventilatory efficiency, and cardiovascular responses during graded exercise. Derived indices—such as peak oxygen uptake (peak VO_2_), VE/VCO_2_ slope, and O_2_ pulse—help localize exercise limitation and are widely used in clinical evaluation and mechanistic research [5].

Both obesity and T2DM are independently associated with impaired CRF; however, their joint influence on exercise physiology is not straightforward. In obesity, absolute peak VO_2_ is strongly influenced by body size and metabolically active tissue, so it may appear preserved even when weight-normalized CRF is substantially reduced. This dissociation points to the dominant contribution of lean (metabolically active) tissue to maximal oxygen uptake during exercise [6]. By contrast, T2DM is consistently associated with reduced exercise tolerance—even without overt cardiovascular disease—reflecting integrated abnormalities in exercise cardiac output, peripheral oxygen extraction, microvascular perfusion, and skeletal muscle metabolism [7, 8]. Prior work has often considered obesity and T2DM in isolation or treated diabetes as a uniform main effect, rather than directly testing whether its functional impact varies with obesity severity. As a result, clinically relevant effect modification may be missed, and apparently paradoxical patterns may be harder to interpret.

Middle age represents an important window in which obesity and T2DM are common, while advanced cardiovascular complications may still be absent or subclinical [9]. Although low CRF (often quantified by peak VO_2_) predicts cardiovascular and all-cause mortality—particularly in diabetes or impaired glucose metabolism—CPX characteristics across obesity–diabetes phenotypes in adults aged 30–50 years remain insufficiently described [10, 11]. Observational comparisons are also vulnerable to confounding because individuals with T2DM often differ systematically from those without diabetes in age, body composition, comorbidities, and metabolic profile. These imbalances can limit causal interpretation when relying on conventional multivariable adjustment alone.

To address these gaps, we conducted a propensity score–weighted analysis of middle-aged adults undergoing CPX to evaluate the independent and joint effects of obesity severity and T2DM on CRF. By explicitly testing for effect modification and integrating CPX indices with body composition and glycemic measures, we sought to clarify how obesity severity shapes the functional consequences of diabetes. We hypothesized that the association between T2DM and CRF would differ across obesity strata, and that body composition and glycemic burden would be key determinants of exercise capacity. This study is reported in accordance with the STROBE guidelines [12].

## Materials and Methods

### Study design and participants

**Figure 1:**
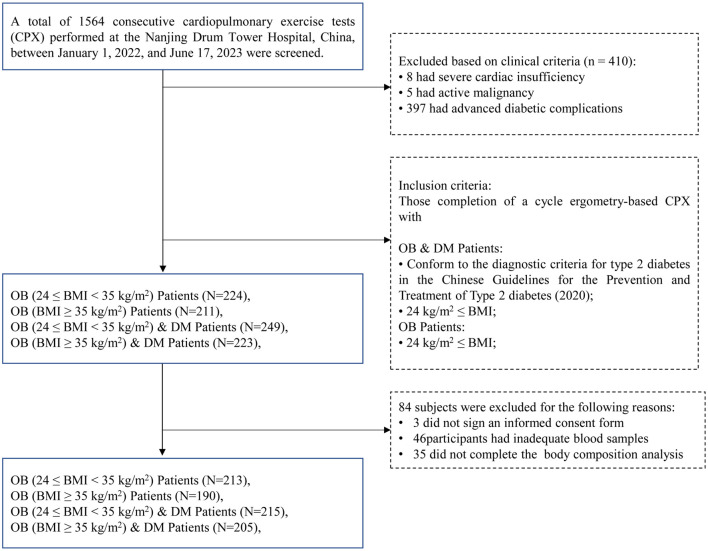
Study enrollment flowchart.

This observational cross-sectional study was conducted at the Drum Tower Clinical Medicine College of Nanjing University of Chinese Medicine between January 1, 2022, and June 17, 2023. Adults who underwent clinically indicated CPX were consecutively screened (Fig. 1). Participants were eligible if they met the following criteria: 1) age between 20 and 50 years; 2) completion of symptom-limited CPX with valid peak effort (respiratory exchange ratio ≥ 1.10 or ≥ 85% of age-predicted maximal heart rate); and 3) availability of relevant anthropometric, laboratory, and clinical data. Participants with severe cardiac insufficiency, active malignancy, or advanced diabetic complications were excluded prior to data completeness screening. Advanced diabetic complications were defined as clinically significant diabetes-related end-organ damage, including diabetic nephropathy (estimated glomerular filtration rate (eGFR) < 30 mL/min/1.73 m^2^), proliferative diabetic retinopathy, diabetic foot with active ulceration, and cardiovascular autonomic neuropathy characterized by abnormal heart rate or blood pressure responses. After exclusion based on predefined clinical criteria (n = 410), 1,154 participants remained eligible.

We restricted the study population to middle-aged adults because CPX requires maximal or near-maximal effort, which may not be feasible or safe in older individuals with multiple comorbidities. This approach is consistent with prior CPX-based clinical and epidemiological studies.

Obesity was defined according to Chinese guidelines and categorized as moderate obesity (24 ≤ BMI < 35 kg/m^2^) or severe obesity (BMI ≥ 35 kg/m^2^) [13]. BMI was used for phenotype classification, and no additional BMI-based inclusion or exclusion criteria were applied. T2DM was identified based on a documented clinical diagnosis or standard diagnostic criteria.

Among the eligible participants, an additional 84 were excluded due to incomplete CPX, laboratory, and body composition data. Therefore, a total of 823 participants were included in the final analysis. Participants were categorized into four phenotypes: moderate obesity without T2DM; severe obesity without T2DM; moderate obesity with T2DM; and severe obesity with T2DM.

The study was approved by the Ethics Committee of the Drum Tower Clinical Medicine College of Nanjing University of Chinese Medicine (Approval No.: 2022-412-02). The study was conducted in compliance with the ethical standards of the responsible institution on human subjects and with the Helsinki Declaration.

### CPX

All participants underwent symptom-limited maximal CPX using a cycle ergometer (Quark PFT Ergo, COSMED, Rome, Italy) following a standardized ramp protocol. After a 3-min rest period and a 3-min warm-up, workload was increased continuously at an individualized rate to achieve volitional exhaustion within 8–12 min [14]. Tests were terminated according to standard American College of Sports Medicine criteria.

Breath-by-breath gas exchange variables were measured and averaged over 10-s intervals. Peak oxygen uptake (VO_2_) was reported as absolute (L/min) and relative (mL/kg/min) values. The anaerobic threshold was determined using the V-slope method. Ventilatory efficiency was assessed using the VE/VCO_2_ slope, calculated from 1 min after exercise onset to the isocapnic buffering period. Maximal effort was defined as a respiratory exchange ratio ≥ 1.10 [15] or achievement of ≥ 85% of age-predicted maximal heart rate.

### Anthropometry and laboratory measurements

Body height and weight were measured using standardized equipment (Inbody720, South Korea), and lean body mass (LBM) was assessed via bioelectrical impedance analysis.

Fasting venous blood samples (5 mL) were collected. Biochemical markers (FPG, 2-hPG, HbA1c, TG, TC, HDL-C, LDL-C) were measured using automated analyzers (Beckman, USA; Tosoh, Japan).

### Statistical analysis

Baseline characteristics and unadjusted CPX variables were summarized using descriptive statistics and compared across groups using one-way analysis of variance (ANOVA) or Kruskal–Wallis tests with appropriate post hoc adjustments.

To address confounding arising from non-random allocation to obesity–diabetes phenotypes, we applied propensity score weighting (PSW) to balance baseline covariates across groups. Propensity scores were estimated via multinomial logistic regression including demographic, anthropometric, and clinical covariates.

To evaluate the interaction between BMI category and T2DM on peak VO_2_, we constructed a series of progressively adjusted PSW-weighted linear regression models. Model 1 included BMI category, T2DM, age, and sex. Model 2 further adjusted for smoking, hypertension, treatment, and diabetes duration. Model 3 additionally included LBM and was used as the final model for primary inference. The statistical significance of the BMI × T2DM interaction term was evaluated using a Wald test.

Missing data were assessed across all collected variables, with proportions ranging from 0% to 52.5%. A detailed missingness table is provided in Supplementary Material 1 (jocmr.elmerjournals.com). Variables with more than 10% missingness were excluded from the primary analyses. For the remaining variables, missing data were handled using multiple imputation with chained equations (MICE), generating five imputed datasets (m = 5) with a random seed of 123, as a sensitivity analysis. The imputation model included all variables used in the primary analyses, including exposure, outcome, and covariates. Given that the variables retained in the primary analyses had relatively low-to-moderate levels of missingness (< 10%) and that multiple imputation was used for sensitivity purposes rather than primary inference, m = 5 imputations were considered sufficient to obtain stable estimates while maintaining computational efficiency. Implausible physiological values were predefined based on clinical criteria and recoded as missing prior to imputation. Sensitivity analyses comparing results from the original (complete-case) and imputed datasets yielded largely consistent findings. Sex was retained as a key covariate despite moderate missingness due to its established clinical relevance.

To address concerns regarding the international applicability of BMI classification, we conducted a prespecified sensitivity analysis using WHO BMI categories. Because a full cross-classification of WHO BMI categories with diabetes status would have resulted in sparse subgroups and unstable estimates, BMI categories were collapsed into three groups: overweight (25.0–29.9 kg/m^2^), obesity class I/II (30.0–39.9 kg/m^2^), and obesity class III (≥ 40.0 kg/m^2^).

All primary analyses, including baseline comparisons, CPX variables, and propensity score–weighted models, were repeated using these WHO-based categories. Results of these sensitivity analyses are presented in Supplementary Materials 2–4 (jocmr.elmerjournals.com).

Predicted marginal means and 95% confidence intervals for each BMI–DM combination were derived from the fitted interaction model. Weighted correlation analyses and weighted linear regression were conducted to identify independent correlates of peak VO_2_. Statistical significance was defined as a two-sided P value < 0.05.

All analyses were performed using R (version 4.5.1; R Foundation for Statistical Computing, Vienna, Austria) and RStudio (version 2025.09.1; Posit Software, Boston, MA, USA). PSW and weighted regression analyses were conducted using the “survey” package, propensity scores were estimated using the “nnet” package, multiple imputation was performed using the “mice” package [16], and data processing was conducted using the “dplyr” and “readxl” packages.

## Results

### Participant characteristics

Table 1 presents baseline demographic, anthropometric, metabolic, clinical, and treatment characteristics across the four phenotypes. Participants with T2DM were older and had higher fasting and postprandial glucose, HbA1c, and triglycerides than non-diabetic participants within the same BMI stratum. Body weight, BMI, and LBM increased progressively with obesity severity irrespective of diabetes status. Treatment use was more frequent among participants with T2DM, as expected, reflecting their underlying cardiometabolic condition.

**Table 1:** Baseline Characteristics of Participants According to Obesity–Diabetes Phenotype

Variable	None DM		DM		P value
	Moderate obesity (24 ≤ BMI < 35) (n = 213)	Severe obesity (BMI ≥ 35) (n = 190)	Moderate obesity (24 ≤ BMI < 35) (n = 215)	Severe obesity (BMI ≥ 35) (n = 205)	
Age (years)	29.8 ± 8.0	28.5 ± 8.3	34.9 ± 10.7	31.7 ± 9.0	< 0.001
Female sex, n (%)	72 (28)	101 (45)	204 (74)	120 (48)	< 0.001
Height (cm)	168.6 ± 9.6	169.4 ± 8.8	169.8 ± 8.7	169.1 ± 8.0	0.49
Body weight (kg)	88.4 ± 11.0	118.3 ± 19.2	82.3 ± 12.5	116.1 ± 18.1	< 0.001
BMI (kg/m^2^)	31.1 ± 2.8	41.1 ± 4.9	28.5 ± 3.0	40.5 ± 4.6	< 0.001
Lean body mass (kg)	53.5 ± 10.3	64.1 ± 12.7	56.7 ± 9.4	63.7 ± 11.8	< 0.001
Duration of diabetes (years)	-	-	3.3 ± 4.5	1.0 ± 2.0	-
Family history of diabetes, n (%)	24 (9)	0 (0)	96 (35)	45 (20)	< 0.001
Fasting plasma glucose (mmol/L)	4.6 ± 0.5	4.8 ± 0.4	8.6 ± 2.9	8.4 ± 3.0	< 0.001
2-h postprandial glucose (mmol/L)	5.9 ± 1.6	7.3 ± 1.6	15.5 ± 4.2	14.0 ± 4.1	< 0.001
HbA1c (%)	5.3 ± 0.3	5.5 ± 0.3	8.9 ± 2.5	8.6 ± 3.9	< 0.001
Triglycerides (mmol/L)	1.5 ± 0.7	1.9 ± 0.8	2.5 ± 2.0	2.6 ± 2.0	< 0.001
Total cholesterol (mmol/L)	4.1 ± 0.8	5.7 ± 0.9	4.6 ± 1.1	5.2 ± 1.0	0.040
HDL cholesterol (mmol/L)	1.2 ± 0.3	1.1 ± 0.3	1.1 ± 0.2	1.0 ± 0.2	< 0.001
LDL cholesterol (mmol/L)	2.4 ± 0.6	2.7 ± 0.8	2.5 ± 0.9	2.9 ± 0.9	0.70
Hypertension, n (%)	48 (18)	78 (35)	124 (45)	130 (58)	< 0.001
Pulmonary diseases, n (%)	148 (57)	155 (70)	196 (71)	105 (47)	< 0.001
Current smoking, n (%)	0 (0)	8 (4)	56 (20)	20 (9)	< 0.001
Treatment (cardiometabolic medications)^a^, n (%)	76 (29)	64 (29)	76 (28)	60 (24)	0.555
β-blocker, n (%)	26 (10)	18 (8)	24 (9)	0 (0)	< 0.001
ACE inhibitor, n (%)	39 (15)	31 (14)	28 (10)	15 (7)	0.018
CCB, n (%)	18 (7)	15 (7)	32 (12)	50 (22)	< 0.001
Diuretic, n (%)	31 (12)	28 (13)	28 (10)	10 (4)	0.015

Values are presented as mean ± standard deviation or number (percentage). P values were calculated using one-way ANOVA for continuous variables and the χ^2^ test for categorical variables. Treatment includes antihypertensive and cardiometabolic medications (β-blockers, ACE inhibitors, CCBs, and diuretics), and does not refer exclusively to antidiabetic therapy. ^a^Participants may receive more than one type of medication; therefore, counts for individual medication categories are not mutually exclusive. ACE: angiotensin-converting enzyme; ANOVA: analysis of variance; BMI: body mass index; CCB: calcium channel blocker; DM: diabetes mellitus; HbA1c: glycated hemoglobin; HDL: high-density lipoprotein; LDL: low-density lipoprotein.

### Cardiopulmonary exercise performance before weighting

Table 2 shows unadjusted differences in CPX performance across phenotypes. Most indices of exercise capacity, gas exchange, and cardiovascular response showed statistically significant differences across groups, with many comparisons reaching P < 0.001 in the original dataset, including peak VO_2_ (absolute and weight-normalized), peak work rate, peak ventilation, VE/VCO_2_ slope, O_2_ pulse, and peak blood pressure. Resting heart rate and resting systolic/diastolic blood pressure also differed across phenotypes (all P < 0.001).

**Table 2:** Unadjusted Cardiopulmonary Exercise Variables Across Obesity–Diabetes Phenotypes

Variable	None DM		DM		P value
	Moderate obesity (24 ≤ BMI < 35) (n = 213)	Severe obesity (BMI ≥ 35) (n = 190)	Moderate obesity (24 ≤ BMI < 35) (n = 215)	Severe obesity (BMI ≥ 35) (n = 205)	
MVV (L/min)	93.5 ± 34.1	103.8 ± 28.2	97.4 ± 30.8	96.0 ± 29.7	0.004
Forced vital capacity (L)	3.6 ± 1.1	3.8 ± 0.9	3.6 ± 0.8	3.5 ± 0.7	< 0.001
FEV_1_ (L)	3.0 ± 0.9	3.1 ± 0.7	2.9 ± 0.7	2.9 ± 0.7	0.004
FEV_1_/FVC (%)	0.8 ± 0.1	0.8 ± 0.1	0.8 ± 0.1	0.8 ± 0.1	0.355
Resting heart rate (beats/min)	93.5 ± 14.1	95.2 ± 12.7	86.0 ± 12.8	96.1 ± 11.5	< 0.001
Resting systolic blood pressure (mm Hg)	122.7 ± 17.1	128.2 ± 21.2	131.2 ± 26.5	138.3 ± 14.8	< 0.001
Resting diastolic blood pressure (mm Hg)	83.8 ± 10.6	85.8 ± 16.1	82.2 ± 12.2	92.6 ± 10.1	< 0.001
Peak RER	1.1 ± 0.1	1.1 ± 0.1	1.1 ± 0.1	1.1 ± 0.1	< 0.001
Peak VO_2_ (L/min)	1.7 ± 0.4	2.0 ± 0.5	1.4 ± 0.4	2.0 ± 0.5	< 0.001
Peak VO_2_ (mL/kg/min)	18.5 ± 3.8	16.9 ± 3.3	17.9 ± 3.5	16.8 ± 3.0	< 0.001
Peak metabolic equivalents (METs)	5.7 ± 1.1	5.1 ± 0.9	5.2 ± 1.0	5.1 ± 0.9	< 0.001
Peak work rate (W)	136.6 ± 37.3	158.2 ± 41.9	125.7 ± 33.8	151.2 ± 40.6	< 0.001
Peak work rate (W/kg)	1.5 ± 0.3	1.3 ± 0.3	1.5 ± 0.3	1.3 ± 0.3	< 0.001
Peak ventilation (L/min)	53.3 ± 12.3	61.0 ± 16.2	48.8 ± 12.8	58.5 ± 15.3	< 0.001
Peak VCO_2_ (L/min)	2.0 ± 0.5	2.3 ± 0.6	1.8 ± 0.4	2.3 ± 0.6	< 0.001
Peak respiratory rate (breaths/min)	32.6 ± 5.6	34.6 ± 8.3	30.1 ± 5.7	34.4 ± 8.3	< 0.001
Breathing reserve (%)	69.3 ± 20.9	67.3 ± 19.5	70.9 ± 20.1	66.7 ± 22.2	0.191
VE/VCO_2_ slope	26.7 ± 2.3	26.3 ± 2.3	27.3 ± 2.9	26.0 ± 2.9	< 0.001
VO_2_ at anaerobic threshold (L/min)	1.1 ± 0.2	1.3 ± 0.3	1.0 ± 0.2	1.3 ± 0.3	< 0.001
Peak O_2_ pulse (mL/beat)	11.1 ± 2.7	14.6 ± 5.1	10.5 ± 2.2	13.4 ± 3.0	< 0.001
ΔVO_2_/ΔWork rate (mL/min/W)	12.3 ± 1.5	12.8 ± 1.5	11.7 ± 1.8	13.2 ± 1.8	< 0.001
Peak heart rate (beats/min)	152.7 ± 15.2	149.6 ± 15.7	144.7 ± 21.4	147.0 ± 12.8	< 0.001
Percent predicted maximal heart rate (%)	80 ± 11	81 ± 14	80 ± 5	80 ± 10	0.001
Peak systolic blood pressure (mm Hg)	176.3 ± 25.8	195.8 ± 26.0	179.4 ± 34.3	193.3 ± 27.7	< 0.001
Peak diastolic blood pressure (mm Hg)	85.1 ± 18.7	89.8 ± 17.9	87.3 ± 17.4	93.2 ± 14.4	< 0.001

Values are presented as mean ± standard deviation. Variables with missing data exceeding 10% (peak respiratory rate, breathing reserve, and peak blood pressure) were not included as covariates in the propensity score weighting models. Missing values were handled using multiple imputation, and sensitivity analyses comparing original and imputed datasets yielded consistent results. P values were calculated using one-way analysis of variance or the Kruskal–Wallis test, as appropriate. P values were calculated using the original dataset. For most variables, results were consistent after multiple imputation. Breathing reserve was the only variable showing discrepant significance (original dataset: P = 0.191; after imputation: P < 0.001) and should therefore be interpreted with caution. Implausible values were identified based on predefined physiological ranges and set to missing prior to analysis. Peak VO_2_: maximal oxygen consumption; RER: respiratory exchange ratio; VE: minute ventilation; VCO_2_: carbon dioxide output; FEV_1_: forced expiratory volume in 1 second; FVC: forced vital capacity; MVV: maximal voluntary ventilation.

Resting pulmonary/ventilatory measures showed only modest between-group differences: maximal voluntary ventilation (MVV, P = 0.004), forced vital capacity (FVC, P < 0.001), and forced expiratory volume in 1 second (FEV_1_, P = 0.004) differed statistically, whereas FEV_1_/FVC was similar across phenotypes (P = 0.355). Breathing reserve was not different in the original dataset (P = 0.191) but differed after multiple imputation (P < 0.001), indicating sensitivity of this comparison to missing-data handling and warranting cautious interpretation.

Within both diabetes strata, severe obesity (≥ 35 kg/m^2^) was associated with higher absolute exercise capacity than moderate obesity (24 to < 35 kg/m^2^), including higher peak VO_2_ (L/min), peak work rate, peak ventilation, peak VCO_2_, O_2_ pulse, and ΔVO_2_/Δwork rate (all P < 0.001).

By contrast, the diabetes-related pattern depended on obesity severity. In moderate obesity, T2DM was associated with lower peak VO_2_, peak work rate, O_2_ pulse, ΔVO_2_/Δwork rate, and peak heart rate compared with non-diabetic counterparts (all P < 0.001). In severe obesity, these diabetes-related differences were attenuated and were no longer evident for several absolute indices; notably, peak VO_2_ did not differ by T2DM status (P = 0.272).

Weight-normalized indices (peak VO_2_ in mL/kg/min, METs, and W/kg) were consistently lower in the high-BMI groups regardless of diabetes status (all P < 0.001), reinforcing a dissociation between absolute capacity and weight-normalized performance.

### PSW-adjusted main effects on peak VO_2_

After weighting, differences in peak VO_2_ remained across phenotypes (Table 3). The lowest PSW-adjusted peak VO_2_ was observed in moderate obesity with T2DM (1.44 ± 0.02 L/min), while both severe-obesity groups showed higher values (1.95 L/min), values which were similar after rounding.

**Table 3:** Propensity Score–Weighted Outcome Analyses: PSW-Adjusted Main Effects on Peak VO_2_ (L/min)

Group	Peak VO_2_ (mean ± SE)
Moderate obesity, no DM	1.69 ± 0.03
Severe obesity, no DM	1.95 ± 0.04
Moderate obesity, DM	1.44 ± 0.02
Severe obesity, DM	1.95 ± 0.03

Data are presented as mean ± standard error (SE), predicted values with 95% confidence intervals, or regression coefficients, as appropriate. All analyses were conducted using propensity score weighting to balance baseline covariates across obesity–diabetes phenotypes. Predicted values were derived from a propensity score–weighted linear regression model including a BMI × diabetes interaction term (P for interaction < 0.001). Predicted values and regression estimates were derived from the same propensity score–weighted linear regression model including BMI category, T2DM status, their interaction term, and all covariates. Medication classes were encoded as separate binary indicators because treatment categories were not mutually exclusive. BMI: body mass index; DM: diabetes mellitus; PSW: propensity score weighting; VO_2_: oxygen uptake. The footnote applies to Tables 3–7.

### BMI × diabetes interaction on predicted peak VO_2_

The BMI-category × T2DM interaction for peak VO_2_ was statistically significant (P for interaction < 0.001). Predicted peak VO_2_ differed markedly by DM status among individuals with moderate obesity, with lower values in those with DM compared with non-DM counterparts (1.12 (95% CI 1.05–1.18) vs. 1.54 (1.50–1.58) L/min). In contrast, predicted peak VO_2_ was similar between diabetic and non-diabetic participants with severe obesity (1.72 (1.67–1.77) vs. 1.74 (1.68–1.80) L/min) (Table 4).

**Table 4:** Propensity Score–Weighted Outcome Analyses: BMI × DM Interaction: Predicted Peak VO_2_ (L/min)

BMI category	Diabetes status	Predicted peak VO_2_ (95% CI)
Moderate obesity	No DM	1.54 (1.50–1.58)
Severe obesity	No DM	1.74 (1.68–1.80)
Moderate obesity	DM	1.12 (1.05–1.18)
Severe obesity	DM	1.72 (1.67–1.77)

In the fully adjusted PSW model (Table 5), T2DM was associated with lower peak VO_2_ (β = −0.2186, P < 0.001), whereas severe obesity alone was not significantly associated with peak VO_2_ (β = 0.0187, P = 0.582). Importantly, the BMI category × T2DM interaction term was positive and statistically significant (β = 0.2785, P < 0.001), indicating that the negative association of T2DM with peak VO_2_ was attenuated at higher levels of obesity.

**Table 5:** Propensity Score–Weighted Outcome Analyses: Full PSW-Adjusted Interaction Model for Peak VO_2_ (L/min)

Term	Estimate (β)	Std. error	95% CI	P value
Intercept	0.3073	0.1308	(0.0509, 0.5637)	0.019
Severe obesity	0.0187	0.0339	(−0.0478, 0.0851)	0.582
T2DM	−0.2186	0.0412	(−0.2993, −0.1379)	< 0.001
Age	−0.0019	0.0014	(−0.0048, 0.0009)	0.186
Sex (male)	−0.0482	0.0523	(−0.1507, 0.0543)	0.357
Smoking	−0.0799	0.0530	(−0.1838, 0.0240)	0.132
Hypertension	0.0009	0.0273	(−0.0526, 0.0544)	0.974
β-blocker use (yes)	−0.0111	0.044	(−0.0974, 0.0752)	0.801
ACEI use (yes)	−0.0121	0.0337	(−0.0781, 0.0539)	0.719
CCB use (yes)	0.0485	0.0293	(−0.0089, 0.1059)	0.098
Diuretic use (yes)	−0.0332	0.033	(−0.1578, 0.0286)	0.062
Diabetes duration	−0.0145	0.0061	(−0.0265, −0.0026)	0.017
Lean body mass	0.0271	0.0025	(0.0222, 0.0320)	< 0.001
Severe obesity × T2DM	0.2785	0.0540	(0.1727, 0.3843)	< 0.001

Consistent with this, the adverse association of T2DM with peak VO_2_ was evident in moderate obesity but largely attenuated in severe obesity.

### Correlates of peak VO_2_ in PSW analyses

In weighted correlation analyses (Table 6), peak VO_2_ was inversely correlated with 2-h postprandial glucose (r = −0.27, P < 0.001) and strongly positively correlated with LBM (r = 0.70, P < 0.001).

**Table 6:** Propensity Score–Weighted Outcome Analyses: Weighted Correlation With Peak VO_2_ (L/min)

Variable	Weighted r	P value
2-h postprandial glucose (mmol/L)	−0.27	< 0.001
Lean body mass (kg)	0.7	< 0.001

These associations were confirmed in weighted linear regression analyses (Table 7) Higher postprandial glucose levels were independently associated with lower peak VO_2_ (β = −0.01, P < 0.001), whereas LBM was a strong positive determinant (β = 0.04, P < 0.001). In contrast, age and sex were not independently associated with peak VO_2_ after weighting.

**Table 7:** Propensity Score–Weighted Outcome Analyses: Weighted Linear Regression for Peak VO_2_ (L/min)

Variable	Estimate	Std. error	t value	P value
Intercept	−0.13	0.09	−1.47	0.143
2-h postprandial glucose (mmol/L)	−0.01	0	−6.63	< 0.001
Lean body mass (kg)	0.04	0	27.21	< 0.001
Age (years)	0	0	−1.55	0.121
Sex (reference: female)	-	-	-	-

LBM emerged as the strongest correlate of peak VO_2_, whereas postprandial glycemic burden showed a consistent inverse association.

### Sensitivity analysis using WHO BMI classification

Sensitivity analyses using WHO BMI classification (Supplementary Materials 2–4, jocmr.elmerjournals.com) yielded results broadly consistent with the primary analyses. The interaction between BMI category and T2DM on peak VO_2_ remained statistically significant, and the overall pattern of attenuation of the diabetes-associated reduction in peak VO_2_ with increasing obesity severity was preserved.

Associations of LBM and postprandial glucose with peak VO_2_ were also consistent in direction and magnitude, supporting the robustness of the main findings across BMI classification systems.

### Summary of PSW findings

Collectively, PSW analyses demonstrate a significant BMI category × T2DM interaction on peak VO_2_, indicating that obesity severity modifies the association between diabetes and CRF. The adverse impact of T2DM was evident in moderate obesity and but largely attenuated in severe obesity. LBM and postprandial glycemic burden emerged as the strongest independent correlates of peak VO_2_, underscoring phenotype-specific determinants of exercise capacity beyond BMI and diabetes status alone.

## Discussion

We used PSW to examine cardiopulmonary exercise capacity across obesity–diabetes phenotypes in middle-aged adults. The key finding was that the diabetes–fitness association differed across obesity severity strata. In moderate obesity (BMI 24 to < 35 kg/m^2^), diabetes was associated with lower peak VO_2_, whereas in severe obesity (BMI ≥ 35 kg/m^2^), this relationship was markedly attenuated. Together, these results indicate that obesity severity modifies how diabetes-related metabolic dysfunction translates into exercise limitation. Although the endpoints in this study were functional CPX-derived measures rather than hard clinical outcomes, peak VO_2_ and CRF are well-established markers of cardiovascular risk and mortality. Therefore, the phenotype-specific differences observed here are likely to have clinical relevance beyond purely physiological differences.

Reduced CRF is a well-established feature of T2DM, even in the absence of overt cardiovascular disease [17, 18]. From a physiological perspective, diabetes-related impairments in aerobic capacity have been linked to alterations in cardiac output, skeletal muscle oxidative metabolism, endothelial function, and microvascular perfusion. Our results agree with prior evidence of reduced CRF in T2DM, while adding a phenotype-specific perspective showing that the magnitude of impairment is not uniform across obesity severity. In individuals with moderate obesity (BMI 24 to < 35 kg/m^2^), diabetes was associated with a substantial reduction in predicted peak VO_2_, whereas in severe obesity (BMI ≥ 35 kg/m^2^), predicted peak VO_2_ was similar between those with and without diabetes. This pattern supports effect modification by obesity severity rather than a uniform main effect of diabetes. The positive interaction term further suggests that the negative association between T2DM and peak VO_2_ is partially offset at higher levels of obesity. Pairwise comparisons further localized this heterogeneity, showing that diabetes-related reductions in absolute exercise capacity were largely confined to individuals with moderate obesity (BMI 24 to < 35 kg/m^2^), whereas corresponding differences were markedly attenuated or no longer evident among those with severe obesity (BMI ≥ 35 kg/m^2^). Importantly, this effect modification would not have been detectable using conventional multivariable adjustment alone, underscoring the value of the propensity score–weighted framework employed in this study.

The strong association between LBM and peak VO_2_ observed in the weighted analyses may provide a plausible physiological explanation for this pattern. Although increasing adiposity is associated with lower weight-normalized aerobic capacity, absolute peak VO_2_ is strongly dependent on the amount of metabolically active tissue. In the present study, LBM emerged as the strongest independent determinant of peak VO_2_, with an effect size far exceeding that of age or sex. This is consistent with prior evidence showing that maximal oxygen uptake is primarily determined by metabolically active tissue and that lean-adjusted CPX measures may provide a more physiologically meaningful assessment of exercise capacity than total body weight alone [6, 19–21]. In this context, the relatively preserved absolute peak VO_2_ in severe obesity—even with diabetes—may reflect the counterbalancing influence of greater lean mass on oxygen delivery and utilization. Consistent with this interpretation, pairwise analyses showed that individuals with severe obesity exhibited higher absolute peak VO_2_ but uniformly lower weight-normalized indices of exercise performance, underscoring a dissociation between absolute capacity and relative efficiency.

Although LBM was strongly associated with peak VO_2_, formal mediation analysis or higher-order interaction modeling (e.g., BMI × T2DM × LBM) was not performed. Therefore, any mechanistic interpretation involving LBM should be considered exploratory and hypothesis-generating, rather than indicative of a confirmed causal pathway.

Beyond aerobic capacity, ventilatory and circulatory responses during exercise also differed across phenotypes. Increasing obesity severity was associated with higher ventilatory demand during maximal exercise, as evidenced by elevated peak ventilation and a higher VE/VCO_2_ slope. In parallel, peak O_2_ pulse increased with obesity severity, suggesting altered circulatory loading conditions rather than isolated ventilatory limitation. Diabetes further modified these responses in moderate obesity (BMI 24 to < 35 kg/m^2^), where pairwise comparisons demonstrated significantly lower O_2_ pulse, reduced ΔVO_2_/Δwork rate, and lower peak heart rate compared with non-diabetic counterparts, consistent with combined circulatory and peripheral limitations. This profile is compatible with prior evidence linking diabetes to autonomic dysfunction, reduced stroke volume reserve, and impaired skeletal muscle oxidative capacity [17, 18], and indicate that diabetes may exacerbate circulatory and peripheral limitations when excess adiposity has not yet become the dominant constraint. Notably, breathing reserve did not differ significantly across phenotypes in the original dataset, although a difference emerged after multiple imputation; therefore, group differences in ventilatory reserve should be interpreted with caution.

Another clinically relevant finding was the inverse association between postprandial glycemia and peak VO_2_. In weighted regression analyses, 2-h postprandial glucose—but not fasting glucose—was independently associated with reduced aerobic capacity. This finding aligns with growing evidence that postprandial glycemic excursions more accurately reflect cumulative metabolic and vascular stress than fasting glucose, and are more closely linked to functional impairment and cardiovascular risk [22–24]. By directly linking postprandial glucose levels to objective reductions in peak VO_2_, these data encourage interpretation beyond diabetes status alone and toward dynamic glycemic exposure as a potentially actionable correlate.

This dissociation between absolute and weight-normalized performance also helps interpret reports of an apparent “obesity paradox” in cardiometabolic populations. Rather than implying a protective effect of excess adiposity, our results suggest that greater lean mass associated with severe obesity may mask diabetes-related impairments in absolute exercise capacity, while relative performance and efficiency remain compromised. This interpretation reconciles apparently paradoxical observations without invoking protective effects of adiposity itself.

Clinically, these phenotype-specific patterns have several implications. CPX interpretation in diabetes should consider obesity severity and body composition rather than assuming a uniform decrement in fitness. Likewise, intervention priorities may differ: improving postprandial glycemia may be particularly relevant in moderate obesity, whereas preserving or augmenting lean mass may be equally important in severe obesity. Although differentiated exercise strategies are increasingly discussed in the context of diabetes management, they have rarely been informed by phenotype-specific CPX data [25, 26].

Several limitations should be acknowledged. First, although treatment status was available and adjusted for in the analyses, detailed information on specific medication classes, dosages, and treatment duration was not uniformly available. In addition, detailed information on diabetes-specific medications (e.g., glucose-lowering agents) was not available in the dataset. Therefore, residual confounding related to heterogeneous medication effects, including antidiabetic treatment, cannot be entirely excluded.

Second, physical activity data were not available in this dataset. Given that habitual physical activity is a major determinant of CRF, residual confounding due to between-group differences in physical activity cannot be excluded. Although the observed interaction between BMI and T2DM remained significant after adjustment for multiple clinical and anthropometric covariates, the potential influence of unmeasured physical activity should be considered when interpreting these findings.

Third, due to the cross-sectional design, we cannot determine whether severe obesity without diabetes represents a stable phenotype or a transitional state, and longitudinal studies are needed to clarify this issue.

Fourth, BMI classification in the primary analysis was based on Chinese guidelines, which may limit direct comparability with studies using WHO criteria. However, sensitivity analyses using WHO BMI categories yielded consistent findings, supporting the robustness and generalizability of the observed patterns across classification systems.

### Conclusion

In middle-aged adults, the adverse association between T2DM and CRF is not uniform but depends strongly on obesity severity. Interpretation of exercise capacity in this setting should therefore incorporate body composition and postprandial glycemic burden, which may also inform phenotype-specific intervention priorities.

## Supplementary Material

Suppl 1 Missingness of variables included in the primary analyses and variables excluded due to high missingness.

Suppl 2 Baseline characteristics according to diabetes status and WHO BMI classification sensitivity grouping.

Suppl 3 Cardiopulmonary exercise testing variables according to diabetes status and WHO BMI classification.

Suppl 4 Propensity score–weighted outcome analyses using WHO BMI classification.

## Data Availability

The data supporting the findings of this study are available from the corresponding author upon reasonable request.

## References

[1] Bluher M (2019). Obesity: global epidemiology and pathogenesis. Nat Rev Endocrinol.

[2] Laukkanen JA, Isiozor NM, Kunutsor SK (2022). Objectively assessed cardiorespiratory fitness and all-cause mortality risk: an updated meta-analysis of 37 cohort studies involving 2,258,029 participants. Mayo Clin Proc.

[3] Ross R, Arena R, Myers J, Kokkinos P, Kaminsky LA (2024). Update to the 2016 American Heart Association cardiorespiratory fitness statement. Prog Cardiovasc Dis.

[4] Ross R, Blair SN, Arena R, Church TS, Despres JP, Franklin BA, Haskell WL (2016). Importance of assessing cardiorespiratory fitness in clinical practice: a case for fitness as a clinical vital sign: a scientific statement from the American Heart Association. Circulation.

[5] Wasserman K, Hansen JE, Sue DY (1987). Principles of exercise testing and interpretation. Journal of Cardiopulmonary Rehabilitation and Prevention.

[6] Ross R, Goodpaster BH, Koch LG, Sarzynski MA, Kohrt WM, Johannsen NM, Skinner JS (2019). Precision exercise medicine: understanding exercise response variability. Br J Sports Med.

[7] Regensteiner JG, Bauer TA, Reusch JE, Quaife RA, Chen MY, Smith SC, Miller TM (2009). Cardiac dysfunction during exercise in uncomplicated type 2 diabetes. Med Sci Sports Exerc.

[8] Phielix E, Mensink M (2008). Type 2 diabetes mellitus and skeletal muscle metabolic function. Physiol Behav.

[9] Hruby A, Hu FB (2015). The epidemiology of obesity: a big picture. Pharmacoeconomics.

[10] Kurl S, Hakkarainen P, Voutilainen A, Lonnroos E (2022). Combined effects of maximal oxygen uptake and glucose status on mortality: The Prospective KIHD cohort study. Scand J Med Sci Sports.

[11] Nesti L, Pugliese NR, Santoni L, Armenia S, Chiriaco M, Sacchetta L, De Biase N (2024). Distinct effects of type 2 diabetes and obesity on cardiopulmonary performance. Diabetes Obes Metab.

[12] von Elm E, Altman DG, Egger M, Pocock SJ, Gotzsche PC, Vandenbroucke JP, Initiative S (2007). The strengthening the reporting of observational studies in epidemiology (STROBE) statement: guidelines for reporting observational studies. Ann Intern Med.

[13] Pan XF, Wang L, Pan A (2021). Epidemiology and determinants of obesity in China. Lancet Diabetes Endocrinol.

[14] Balady GJ, Arena R, Sietsema K, Myers J, Coke L, Fletcher GF, Forman D (2010). Clinician's Guide to cardiopulmonary exercise testing in adults: a scientific statement from the American Heart Association. Circulation.

[15] Guazzi M, Adams V, Conraads V, Halle M, Mezzani A, Vanhees L, Arena R (2012). EACPR/AHA Scientific Statement. Clinical recommendations for cardiopulmonary exercise testing data assessment in specific patient populations. Circulation.

[16] Van Buuren S, Groothuis-Oudshoorn K (2011). mice: Multivariate imputation by chained equations in R. Journal of statistical software.

[17] Montero D, Diaz-Canestro C, Oberholzer L, Lundby C (2019). The role of blood volume in cardiac dysfunction and reduced exercise tolerance in patients with diabetes. Lancet Diabetes Endocrinol.

[18] Nesti L, Pugliese NR, Sciuto P, Natali A (2020). Type 2 diabetes and reduced exercise tolerance: a review of the literature through an integrated physiology approach. Cardiovasc Diabetol.

[19] Zhou N (2021). Assessment of aerobic exercise capacity in obesity, which expression of oxygen uptake is the best?. Sports Med Health Sci.

[20] Osman AF, Mehra MR, Lavie CJ, Nunez E, Milani RV (2000). The incremental prognostic importance of body fat adjusted peak oxygen consumption in chronic heart failure. J Am Coll Cardiol.

[21] Lavie CJ, Milani RV, Mehra MR (2004). Peak exercise oxygen pulse and prognosis in chronic heart failure. Am J Cardiol.

[22] Roberts CK, Little JP, Thyfault JP (2013). Modification of insulin sensitivity and glycemic control by activity and exercise. Med Sci Sports Exerc.

[23] Hershon KS, Hirsch BR, Odugbesan O (2019). Importance of postprandial glucose in relation to A1C and cardiovascular disease. Clin Diabetes.

[24] Takao T, Suka M, Yanagisawa H, Kasuga M (2021). Thresholds for postprandial hyperglycemia and hypertriglyceridemia associated with increased mortality risk in type 2 diabetes patients: A real-world longitudinal study. J Diabetes Investig.

[25] Slebe R, Wenker E, Schoonmade LJ, Bouman EJ, Blondin DP, Campbell DJT, Carpentier AC (2024). The effect of preprandial versus postprandial physical activity on glycaemia: Meta-analysis of human intervention studies. Diabetes Res Clin Pract.

[26] Kobayashi Y, Long J, Dan S, Johannsen NM, Talamoa R, Raghuram S, Chung S (2023). Strength training is more effective than aerobic exercise for improving glycaemic control and body composition in people with normal-weight type 2 diabetes: a randomised controlled trial. Diabetologia.

