# Supplementary material for: Obesity Severity Differentially Shapes Diabetes-Related Impairment in Cardiorespiratory Fitness: A Cross-Sectional Propensity Score–Weighted Analysis of Middle-Aged Adults
Source: J Clin Med Res. 2026 May 31;18(5):326–35. doi: 10.14740/jocmr6519 (PMC13278680; doi:10.14740/jocmr6519)
Supplement: Suppl 1 — Missingness of variables included in the primary analyses and variables excluded due to high missingness. [file jocmr-18-05-326-s001.docx]

**Suppl 1. Missingness of variables included in the primary analyses and variables excluded due to high missingness**

| **Included variables (≤10% missingness)** | **Missing, n (%)** |
| --- | --- |
| Age (years) | 0 (0) |
| Female sex | 0 (0) |
| Height (cm) | 24 (2.9) |
| Body weight (kg) | 24 (2.9) |
| Body mass index (kg/m²) | 0 (0) |
| Lean body mass (kg) | 0 (0) |
| Duration of diabetes (years) | 14 (1.7) |
| Family history of diabetes, n (%) | 17 (2.1) |
| Fasting plasma glucose (mmol/L) | 18 (2.2) |
| 2-h postprandial glucose (mmol/L) | 25 (3) |
| HbA1c (%) | 23 (2.8) |
| Triglycerides (mmol/L) | 34 (4.1) |
| Total cholesterol (mmol/L) | 16 (1.9) |
| HDL cholesterol (mmol/L) | 38 (4.6) |
| LDL cholesterol (mmol/L) | 42 (5.1) |
| Hypertension | 21 (2.6) |
| Pulmonary diseases, n (%) | 36 (4.4) |
| Current smoking | 32 (3.9) |
| Treatment | 27 (3.3) |
| Maximal voluntary ventilation (L/min) | 43 (5.2) |
| Forced vital capacity (L) | 43 (5.2) |
| FEV1 (L) | 35 (4.3) |
| FEV1/FVC (%) | 41 (5) |
| Resting heart rate (beats/min) | 0 (0) |
| Resting systolic blood pressure (mm Hg) | 65 (7.9) |
| Resting diastolic blood pressure (mm Hg) | 65 (7.9) |
| Peak respiratory exchange ratio | 0 (0) |
| Peak VO₂(L/min) | 0 (0) |
| Peak VO₂ (ml/kg/min) | 0 (0) |
| Peak metabolic equivalents (METs) | 0 (0) |
| Peak work rate (watts) | 0 (0) |
| Peak work rate (watts/kg) | 0 (0) |
| Peak ventilation (L/min) | 0 (0) |
| Peak VCO2 (L/min) | 0 (0) |
| Peak respiratory rate (breaths/min) | 36 (4.4) |
| Breathing reserve (%) | 29 (3.5) |
| VE/VCO2 slope | 0 (0) |
| VO2 at anaerobic threshold (L/min) | 0 (0) |
| Peak O2 pulse (ml/beat) | 0 (0) |
| ΔVO2/ΔWork rate (ml/min/watt) | 0 (0) |
| Peak heart rate (beats/min) | 0 (0) |
| Percent predicted maximal heart rate (%) | 0 (0) |
| Peak systolic blood pressure (mm Hg) | 31 (3.8) |
| Peak diastolic blood pressure (mm Hg) | 31 (3.8) |
|  |  |
| **Variables excluded due to high missingness (>10%)** | **Missing, n (%)** |
| FVC | 108 (13.1) |
| Power@AT(watt) | 161 (19.6) |
| Peak VE(L/min) | 161 (19.6) |
| Peak Mets | 108 (13.1) |
| Variables with more than 10% missing data were excluded from the primary analyses. Missing values are presented as number (percentage). | |
