# Supplementary material for: Obesity Severity Differentially Shapes Diabetes-Related Impairment in Cardiorespiratory Fitness: A Cross-Sectional Propensity Score–Weighted Analysis of Middle-Aged Adults
Source: J Clin Med Res. 2026 May 31;18(5):326–35. doi: 10.14740/jocmr6519 (PMC13278680; doi:10.14740/jocmr6519)
Supplement: Suppl 2 — Baseline characteristics according to diabetes status and WHO BMI classification sensitivity grouping. [file jocmr-18-05-326-s002.docx]

**Suppl 2. Baseline characteristics according to diabetes status and WHO BMI classification sensitivity grouping**

| **Variable** | **Overweight - None DM** | **Overweight - DM** | **Obesity I/II - None DM** | **Obesity I/II - DM** | **Obesity III - None DM** | **Obesity III - DM** | **P Value** |
| --- | --- | --- | --- | --- | --- | --- | --- |
| Age (years) | 32.9 ± 8.2 | 34.6 ± 10.5 | 28.2 ± 7.8 | 33.1 ± 9.5 | 28.7 ± 8.4 | 31.5 ± 10.2 | P < 0.001 |
| Height (cm) | 171.6 ± 10.4 | 170.2 ± 9.5 | 168.2 ± 8.4 | 168.9 ± 8.4 | 168.9 ± 9.9 | 170.0 ± 7.6 | P = 0.042 |
| Body weight (kg) | 82.1 ± 12.0 | 79.7 ± 10.5 | 98.2 ± 12.9 | 101.3 ± 12.8 | 127.4 ± 20.8 | 130.9 ± 16.4 | P < 0.001 |
| Body mass index (kg/m²) | 27.8 ± 1.6 | 27.4 ± 1.4 | 34.6 ± 2.7 | 35.4 ± 2.9 | 44.4 ± 4.7 | 45.2 ± 3.7 | P < 0.001 |
| Lean body mass (kg) | 54.6 ± 12.6 | 56.8 ± 9.2 | 56.2 ± 10.6 | 59.8 ± 10.3 | 66.7 ± 13.8 | 68.5 ± 12.2 | P < 0.001 |
| Duration of diabetes (years) | - | 3.8 ± 4.3 | - | 1.1 ± 1.9 | - | 1.2 ± 2.7 |  |
| Fasting plasma glucose (mmol/L) | 4.4 ± 0.6 | 8.3 ± 2.8 | 4.7 ± 0.4 | 8.6 ± 3.0 | 4.9 ± 0.4 | 8.9 ± 3.5 | P < 0.001 |
| 2-h postprandial glucose (mmol/L) | 4.7 ± 0.8 | 15.5 ± 4.3 | 6.8 ± 1.6 | 14.6 ± 4.3 | 7.2 ± 1.5 | 13.9 ± 4.3 | P < 0.001 |
| HbA1c (%) | 5.2 ± 0.3 | 8.9 ± 2.8 | 5.4 ± 0.3 | 9.1 ± 4.1 | 5.6 ± 0.3 | 8.1 ± 1.4 | P < 0.001 |
| Triglycerides (mmol/L) | 1.2 ± 0.6 | 2.8 ± 2.2 | 1.8 ± 0.8 | 2.5 ± 1.8 | 1.8 ± 0.7 | 2.7 ± 2.3 | P < 0.001 |
| Total cholesterol (mmol/L) | 4.8 ± 0.8 | 5.0 ± 1.1 | 4.7 ± 0.8 | 5.1 ± 1.1 | 4.9 ± 0.9 | 4.8 ± 1.0 | P = 0.001 |
| HDL cholesterol (mmol/L) | 1.4 ± 0.3 | 1.1 ± 0.2 | 1.1 ± 0.3 | 1.0 ± 0.2 | 1.0 ± 0.3 | 1.1 ± 0.3 | P < 0.001 |
| LDL cholesterol (mmol/L) | 2.8 ± 0.6 | 2.9 ± 0.8 | 2.9 ± 0.7 | 3.1 ± 0.9 | 3.0 ± 0.8 | 2.8 ± 0.9 | P = 0.009 |
| Female sex, n (%) | 40 (45%) | 124 (82%) | 76 (27%) | 117 (50%) | 57 (50%) | 55 (55%) | P < 0.001 |
| Have a family history of diabetes, n (%) | 24 (27%) | 52 (34%) | 0 (0%) | 60 (28%) | 0 (0%) | 15 (15%) | P < 0.001 |
| Hypertension, n (%) | 16 (18%) | 66 (43%) | 60 (21%) | 114 (54%) | 50 (43%) | 60 (60%) | P < 0.001 |
| Pulmonary diseases, n (%) | 40 (45%) | 120 (79%) | 188 (67%) | 123 (58%) | 75 (65%) | 30 (30%) | P < 0.001 |
| Current smoking, n (%) | 0 (0%) | 34 (22%) | 4 (1%) | 19 (9%) | 4 (3%) | 15 (15%) | P < 0.001 |
| Treatment, n (%)ᵃ | 25 (28.4%) | 42 (27.6%) | 84 (30%) | 61 (25.8%) | 31 (27%) | 25 (25%) | P < 0.001 |
| β-blocker, n (%) | 8 (9%) | 14 (9%) | 28 (10%) | 6 (3%) | 8 (7%) | 0 (0%) | P = 0.002 |
| ACEI, n (%) | 14 (16%) | 18 (12%) | 40 (14%) | 14 (7%) | 16 (14%) | 5 (5%) | P = 0.018 |
| CCB, n (%) | 4 (5%) | 14 (9%) | 23 (8%) | 48 (23%) | 6 (5%) | 20 (20%) | P < 0.001 |
| Diuretic, n (%) | 10 (11%) | 20 (13%) | 36 (13%) | 6 (3%) | 13 (11%) | 10 (10%) | P = 0.005 |

Values are presented as mean ± standard deviation (SD) for continuous variables and number (percentage) for categorical variables. P values were calculated using one-way analysis of variance (ANOVA) for continuous variables and the χ² test for categorical variables. BMI categories were defined according to World Health Organization (WHO) criteria as overweight (25.0–29.9 kg/m²), obesity class I/II (30.0–39.9 kg/m²), and obesity class III (≥40.0 kg/m²). Diabetes duration was defined as 0 years in participants without diabetes. Treatment includes β-blockers, ACE inhibitors (ACEI), calcium channel blockers (CCB), and diuretics.

ᵃ Participants may receive more than one type of medication; therefore, counts for individual medication categories are not mutually exclusive.
