# Supplementary material for: Obesity Severity Differentially Shapes Diabetes-Related Impairment in Cardiorespiratory Fitness: A Cross-Sectional Propensity Score–Weighted Analysis of Middle-Aged Adults
Source: J Clin Med Res. 2026 May 31;18(5):326–35. doi: 10.14740/jocmr6519 (PMC13278680; doi:10.14740/jocmr6519)
Supplement: Suppl 3 — Cardiopulmonary exercise testing variables according to diabetes status and WHO BMI classification. [file jocmr-18-05-326-s003.docx]

**Suppl 3. Cardiopulmonary exercise testing variables according to diabetes status and WHO BMI classification**

| Variable | Overweight - No DM | Overweight - DM | Obesity I/II - No DM | Obesity I/II - DM | Obesity III - No DM | Obesity III - DM | P value |
| --- | --- | --- | --- | --- | --- | --- | --- |
| Maximal voluntary ventilation (L/min) | 105.9 ± 48.9 | 96.7 ± 31.3 | 95.2 ± 26.2 | 99.0 ± 33.3 | 100.2 ± 26.4 | 89.0 ± 19.3 | P = 0.002 |
| Forced vital capacity (FVC, L) | 4.0 ± 1.5 | 3.6 ± 0.9 | 3.6 ± 0.8 | 3.5 ± 0.8 | 3.8 ± 1.0 | 3.4 ± 0.7 | P = 0.006 |
| Forced expiratory volume in 1 second (FEV1, L) | 3.2 ± 1.3 | 3.0 ± 0.8 | 3.0 ± 0.6 | 2.9 ± 0.7 | 3.1 ± 0.8 | 2.8 ± 0.5 | P = 0.002 |
| FEV1/FVC (%) | 0.8 ± 0.1 | 0.8 ± 0.1 | 0.8 ± 0.1 | 0.8 ± 0.1 | 0.8 ± 0.1 | 0.8 ± 0.1 | P = 0.007 |
| Resting heart rate (beats/min) | 89.4 ± 11.1 | 86.0 ± 12.3 | 96.1 ± 13.5 | 91.3 ± 11.3 | 93.6 ± 14.2 | 101.7 ± 11.2 | P < 0.001 |
| Resting systolic blood pressure (mm Hg) | 115.9 ± 14.9 | 124.0 ± 15.1 | 126.8 ± 18.5 | 133.3 ± 16.1 | 131.3 ± 14.8 | 141.7 ± 12.9 | P < 0.001 |
| Resting diastolic blood pressure (mm Hg) | 82.7 ± 8.0 | 80.6 ± 11.2 | 84.4 ± 11.7 | 90.7 ± 12.1 | 90.0 ± 14.8 | 92.4 ± 9.1 | P < 0.001 |
| Peak respiratory exchange ratio (RER) | 1.1 ± 0.0 | 1.1 ± 0.1 | 1.1 ± 0.1 | 1.1 ± 0.1 | 1.1 ± 0.1 | 1.0 ± 0.1 | P < 0.001 |
| Peak VO2 (L/min) | 1.6 ± 0.5 | 1.4 ± 0.4 | 1.8 ± 0.4 | 1.7 ± 0.4 | 2.1 ± 0.5 | 2.1 ± 0.5 | P < 0.001 |
| Peak VO2 (ml/kg/min) | 18.5 ± 3.9 | 18.4 ± 3.6 | 18.0 ± 3.5 | 17.0 ± 3.2 | 16.6 ± 3.5 | 16.2 ± 2.8 | P < 0.001 |
| Peak metabolic equivalents (METs) | 5.6 ± 1.0 | 5.2 ± 1.1 | 5.6 ± 1.1 | 5.1 ± 0.9 | 5.0 ± 1.0 | 4.9 ± 0.9 | P < 0.001 |
| Peak work rate (watts) | 134.3 ± 42.0 | 124.9 ± 36.2 | 144.0 ± 36.7 | 139.3 ± 37.9 | 162.1 ± 45.3 | 161.7 ± 40.0 | P < 0.001 |
| Peak work rate (watts/kg) | 1.6 ± 0.3 | 1.6 ± 0.4 | 1.5 ± 0.3 | 1.4 ± 0.3 | 1.3 ± 0.3 | 1.2 ± 0.2 | P < 0.001 |
| Peak ventilation (VE, L/min) | 50.5 ± 14.9 | 48.6 ± 12.8 | 55.7 ± 11.7 | 55.2 ± 15.5 | 64.8 ± 17.8 | 60.0 ± 13.9 | P < 0.001 |
| Peak VCO2 (L/min) | 1.9 ± 0.6 | 1.8 ± 0.4 | 2.1 ± 0.5 | 2.1 ± 0.5 | 2.4 ± 0.6 | 2.4 ± 0.6 | P < 0.001 |
| Peak respiratory rate (breaths/min) | 31.1 ± 6.0 | 29.6 ± 5.9 | 33.4 ± 6.0 | 31.7 ± 7.8 | 36.0 ± 9.1 | 37.2 ± 7.1 | P < 0.001 |
| Breathing reserve (%) | 78.3 ± 22.3 | 74.2 ± 21.7 | 67.4 ± 19.7 | 65.1 ± 19.6 | 63.1 ± 17.1 | 68.7 ± 24.5 | P < 0.001 |
| VE/VCO2 slope | 26.5 ± 1.9 | 27.5 ± 3.0 | 26.4 ± 2.4 | 26.8 ± 3.0 | 26.6 ± 2.4 | 25.3 ± 2.5 | P < 0.001 |
| VO2 at anaerobic threshold (L/min) | 1.0 ± 0.2 | 1.0 ± 0.2 | 1.1 ± 0.2 | 1.2 ± 0.3 | 1.4 ± 0.3 | 1.4 ± 0.3 | P < 0.001 |
| Peak O2 pulse (ml/beat) | 10.4 ± 2.8 | 10.6 ± 2.3 | 12.5 ± 4.2 | 12.1 ± 2.9 | 15.1 ± 4.6 | 14.2 ± 2.9 | P < 0.001 |
| ΔVO2/ΔWork rate (ml/min/watt) | 11.6 ± 1.3 | 11.7 ± 1.9 | 12.6 ± 1.5 | 12.6 ± 1.5 | 13.2 ± 1.4 | 13.4 ± 2.3 | P < 0.001 |
| Peak heart rate (beats/min) | 148.7 ± 13.9 | 134.7 ± 18.5 | 153.1 ± 15.7 | 144.9 ± 14.7 | 149.0 ± 15.7 | 148.6 ± 11.9 | P < 0.001 |
| Percent predicted maximal heart rate (%) | 0.8 ± 0.1 | 0.7 ± 0.1 | 0.8 ± 0.1 | 0.8 ± 0.1 | 0.8 ± 0.1 | 0.8 ± 0.1 | P < 0.001 |
| Peak systolic blood pressure (mm Hg) | 167.2 ± 23.9 | 175.7 ± 31.8 | 186.0 ± 26.9 | 192.5 ± 30.0 | 200.6 ± 22.5 | 190.2 ± 24.3 | P < 0.001 |
| Peak diastolic blood pressure (mm Hg) | 86.6 ± 20.0 | 86.1 ± 19.1 | 85.7 ± 16.7 | 92.4 ± 14.9 | 91.8 ± 21.4 | 94.4 ± 13.6 | P < 0.001 |

Values are presented as mean ± standard deviation (SD). P values were calculated using one-way analysis of variance (ANOVA).

BMI categories were defined according to World Health Organization (WHO) criteria as overweight (25.0–29.9 kg/m²), obesity class I/II (30.0–39.9 kg/m²), and obesity class III (≥40.0 kg/m²).

VO₂, oxygen uptake; VCO₂, carbon dioxide production; VE, minute ventilation; RER, respiratory exchange ratio; METs, metabolic equivalents; HR, heart rate; SBP, systolic blood pressure; DBP, diastolic blood pressure; AT, anaerobic threshold.
