# Supplementary material for: Obesity Severity Differentially Shapes Diabetes-Related Impairment in Cardiorespiratory Fitness: A Cross-Sectional Propensity Score–Weighted Analysis of Middle-Aged Adults
Source: J Clin Med Res. 2026 May 31;18(5):326–35. doi: 10.14740/jocmr6519 (PMC13278680; doi:10.14740/jocmr6519)
Supplement: Suppl 4 — Propensity score–weighted outcome analyses using WHO BMI classification. [file jocmr-18-05-326-s004.docx]

**Suppl 4. Propensity score–weighted outcome analyses using WHO BMI classification**

| A. PSW-adjusted main effects on peak VO₂(L/min) | | | | | | | | | |  | | | | | | | |
| --- | --- | --- | --- | --- | --- | --- | --- | --- | --- | --- | --- | --- | --- | --- | --- | --- | --- |
|  | | **BMI category** | | | **Diabetes status** | | | | | **n** | | | **Peak VO₂ (mean ± SE)** | | | | |
|  | | Overweight | | | No DM | | | | | 88 | | | 1.59 ± 0.06 | | | | |
|  | | Obesity I/II | | | No DM | | | | | 280 | | | 1.83 ± 0.03 | | | | |
|  | | Obesity III | | | No DM | | | | | 115 | | | 2.02 ± 0.06 | | | | |
|  | | Overweight | | | DM | | | | | 152 | | | 1.52 ± 0.03 | | | | |
|  | | Obesity I/II | | | DM | | | | | 211 | | | 1.63 ± 0.05 | | | | |
|  | | Obesity III | | | DM | | | | | 100 | | | 2.09 ± 0.03 | | | | |
|  | |  | | | | | | | | | | | | | | | |
| B. Adjusted predicted peak VO₂ (L/min) by BMI category and diabetes status (Model 3) | | | | | | | | | | | | | | | | | |
|  | | **BMI category** | **Diabetes status** | | | | | **Predicted peak VO2 (95% CI)** | | | | | |  | | | |
|  | | Overweight | No DM | | | | | 1.76 (1.65–1.88) | | | | | |  | | | |
|  | | Obesity I/II | No DM | | | | | 1.92 (1.84–2.00) | | | | | |  | | | |
|  | | Obesity III | No DM | | | | | 2.05 (1.96–2.15) | | | | | |  | | | |
|  | | Overweight | DM | | | | | 1.77 (1.62–1.92) | | | | | |  | | | |
|  | | Obesity I/II | DM | | | | | 1.76 (1.68–1.83) | | | | | |  | | | |
|  | | Obesity III | DM | | | | | 2.02 (1.95–2.10) | | | | | |  | | | |
|  | | P for interaction (BMI × DM): Model 1 = 0.002; Model 2 = 0.016; Model 3 = 0.017 | | | | | | | | | | | | | | | |
| C. PSW-adjusted interaction model for peak VO₂ (L/min) | | | | | | | | | | | | | | | | | |
|  | **Term** | | | **Estimate (β)** | | | **Std. Error** | | | | **95% CI** | | | | **P value** | | |
|  | Intercept | | | -0.2384 | | | 0.1624 | | | | -0.5566 to 0.0799 | | | | 0.142 | | |
|  | BMI (Obesity I/II) | | | 0.1549 | | | 0.0402 | | | | 0.0762 to 0.2336 | | | | <0.001 | | |
|  | BMI (Obesity III) | | | 0.2888 | | | 0.0565 | | | | 0.1781 to 0.3996 | | | | <0.001 | | |
|  | T2DM | | | 0.0062 | | | 0.0607 | | | | -0.1128 to 0.1252 | | | | 0.919 | | |
|  | Age (years) | | | 0.0029 | | | 0.002 | | | | -0.0011 to 0.0068 | | | | 0.153 | | |
|  | Sex (male) | | | -0.1983 | | | 0.0648 | | | | -0.3253 to -0.0714 | | | | 0.002 | | |
|  | Smoking | | | -0.2572 | | | 0.0789 | | | | -0.4119 to -0.1025 | | | | 0.001 | | |
|  | Hypertension | | | -0.0031 | | | 0.0288 | | | | -0.0596 to 0.0533 | | | | 0.914 | | |
|  | DM duration | | | -0.0255 | | | 0.0073 | | | | -0.0398 to -0.0113 | | | | <0.001 | | |
|  | Lean body mass | | | 0.0327 | | | 0.003 | | | | 0.0267 to 0.0386 | | | | <0.001 | | |
|  | BMI (Obesity I/II) × T2DM | | | -0.1682 | | | 0.0701 | | | | -0.3057 to -0.0308 | | | | 0.017 | | |
|  | BMI (Obesity III) × T2DM | | | -0.036 | | | 0.0779 | | | | -0.1888 to 0.1167 | | | | 0.644 | | |
|  |  | | |  | | |  | | | |  | | | |  | | |
|  | | | | | | | | | |  | | | | | | | |
| D. Weighted correlation with peak VO₂(L/min) | | | | | | | | | |  | | | | | | | |
|  | | Variable | | | Weighted r | | | | | P value | | | | | |  | |
|  | | 2-h postprandial glucose (mmol/L) | | | -0.21 | | | | | <0.001 | | | | | |  | |
|  | | Lean body mass (kg) | | | 0.61 | | | | | <0.001 | | | | | |  | |
|  | |  | | |  | | | | |  | | | | | |  | |
| E. Weighted linear regression for peak VO₂(L/min) | | | | | | | | | |  | | | | | | | |
|  | | **Variable** | | | | **Estimate** | | | **Std. Error** | | | **t value** | | **P value** | | |  |
|  | | Intercept | | | | -0.1985 | | | 0.1569 | | | -1.27 | | 0.206 | | |  |
|  | | 2-h postprandial glucose (mmol/L) | | | | -0.021 | | | 0.0035 | | | -6.08 | | <0.001 | | |  |
|  | | Lean body mass (kg) | | | | 0.0385 | | | 0.0025 | | | 15.39 | | <0.001 | | |  |
|  | | Age (years) | | | | 0.002 | | | 0.0024 | | | 0.83 | | 0.405 | | |  |
|  | | Sex (male) | | | | -0.3504 | | | 0.0577 | | | -6.07 | | <0.001 | | |  |
|  | |  | | | |  | | |  | | |  | |  | | |  |
| Values are presented as mean ± standard error (SE), predicted values with 95% confidence intervals, or regression coefficients, as appropriate. All analyses were conducted using propensity score weighting (PSW).  Predicted values and regression estimates were derived from PSW-adjusted linear regression models including BMI category, diabetes status, their interaction, and covariates.  Model 1 was adjusted for age and sex; Model 2 was additionally adjusted for smoking status, hypertension, treatment status, and diabetes duration; Model 3 was further adjusted for lean body mass.  Treatment status was included as a covariate in the propensity score and regression models but is not shown in the final model due to non-significance.  BMI categories were defined according to World Health Organization (WHO) criteria.  VO₂, oxygen uptake; DM, diabetes mellitus. | | | | | | | | | | | | | | | | | |
